# General Practitioners’ Perspective on eHealth and Lifestyle Change: Qualitative Interview Study

**DOI:** 10.2196/mhealth.8988

**Published:** 2018-04-17

**Authors:** Carl Joakim Brandt, Gabrielle Isidora Søgaard, Jane Clemensen, Jens Sndergaard, Jesper Bo Nielsen

**Affiliations:** ^1^ Research Unit of General Practice Department of Public Health University of Southern Denmark Odense Denmark; ^2^ Centre for Innovative Medical Technology University of Southern Denmark Odense Denmark; ^3^ Hans Christian Andersen’s Childrens Hospital Odense University Hospital Odense Denmark

**Keywords:** general practice, primary care, technology, motivational interviewing, telemedicine, mentoring

## Abstract

**Background:**

Wearables, fitness apps, and patient home monitoring devices are used increasingly by patients and other individuals with lifestyle challenges. All Danish general practitioners (GPs) use digital health records and electronic health (eHealth) consultations on a daily basis, but how they perceive the increasing demand for lifestyle advice and whether they see eHealth as part of their lifestyle support should be explored further.

**Objective:**

This study aimed to explore GPs’ perspectives on eHealth devices and apps and the use of eHealth in supporting healthy lifestyle behavior for their patients and themselves.

**Methods:**

A total of 10 (5 female and 5 male) GPs were recruited by purposive sampling, aged 38 to 69 years (mean 51 years), of which 4 had an urban uptake of patients and 6 a rural uptake. All of them worked in the region of Southern Denmark where GPs typically work alone or in partnership with 1 to 4 colleagues and all use electronic patient health records for prescription, referral, and asynchronous electronic consultations. We performed qualitative, semistructured, individual in-depth interviews with the GPs in their own office about how they used eHealth and mHealth devices to help patients challenged with lifestyle issues and themselves. We also interviewed how they treated lifestyle-challenged patients in general and how they imagined eHealth could be used in the future.

**Results:**

All GPs had smartphones or tablets, and everyone communicated on a daily basis with patients about disease and medicine via their electronic health record and the internet. We identified 3 themes concerning the use of eHealth: (1) how eHealth is used for patients; (2) general practitioners’ own experience with improving lifestyle and eHealth support; and (3) relevant coaching techniques for transformation into eHealth.

**Conclusions:**

GPs used eHealth frequently for themselves but only infrequently for their patients. GPs are familiar with behavioral change techniques and are ready to use them in eHealth if they are used to optimize processes and not hinder other treatments. Looking ahead, education of GPs and recognizing patients’ ability and preference to use eHealth with regard to a healthy living are needed.

## Introduction

Wearables, fitness apps, and patient home monitoring devices are increasingly used by the general population. A recent study showed that 96% of a representative sample of 1004 Danes between 40 and 60 years prefers lifestyle change to medication [1], and outcomes for patients engaging in medical treatment has shown to be significantly affected by patients’ engagement [2].

Internet and mobile interventions have the ability to improve lifestyle behaviors for patients when behavior change elements are used by health professionals [3]. However, teams with both medical doctors and lifestyle coaches did not perform better than teams without medical doctors [4]. General practitioners (GPs) are central in the Danish health care system and have specialization certificates equal to other specialties for medical doctors. Close to 85% of all Danes see a GP at least once annually [5], and the GP is the patient’s primary contact point to the health care system. The GPs have a 5-year postgraduate specializing degree and act as gatekeepers between the primary levels and the specialized health care system, covering hospitals, private hospitals, and specialists.

We have previously described a collaborative electronic health (eHealth) solution that supported lifestyle coaching by establishing a relationship and providing behavioral change (weight loss of 7.0 kg for 20 months) through monitoring and empathic, relevant, and individualized feedback in a general practice setting [6]. These findings were replicated in a later version of the solution in a municipality setting in the region of North East England in Durham and Darlington County for men with type 2 diabetes in a pilot randomized controlled trial where patients lost an average of 5.4 kg compared with a control group that received usual care and lost 2.8 kg. In that same study, in-depth interviews with participating patients revealed that meeting face to face was important for the patients [7], and a recent qualitative study has shown that building a relationship to a health care professional using collaborative eHealth for lifestyle change is probably the most important driver for successful long-term outcome [8].

The role of GPs with regard to support of patients with lifestyle challenges and use of eHealth has not yet been explored. Hence, we aimed to identify factors important to GPs assisting patients undergoing lifestyle changes. Of particular focus was how the GPs see eHealth as a part of their own and their patients’ struggle to live a healthier life.

## Methods

### Context

We performed semistructured in-depth individual interviews with 10 GPs in the Region of Southern Denmark. In 2017, there were approximately 3500 GPs in Denmark covered by the collective agreement with the public health care system. On average, each GP had 1600 patients. In the Region of Southern Denmark, there were 786 full-time employed GPs working in 378 shared or solo practices [5]. GPs were paid partly by a per capita remuneration (30%) and partly a fee for service offering (70%) such as fees for consultations (20 €), telephone consultations (3,5 €), asynchronous e-consultations (6 €), various blood tests, and other relevant GP tasks. GPs did not receive remuneration for receiving or interpreting patient-registered outcome measurements (PROMs). Patients were generally quite loyal to their GP and change doctor rarely.

### Sampling

Purposeful sampling was conducted comprising 10 GPs recruited by email or phone. The criteria were gender, seniority, age, registration as sole practitioner or in a shared medical practice, and patient recruitment area. In total, 11 GPs were invited and 1 declined to participate. After 9 interviews, no new themes or subthemes emerged, and an additional tenth interview confirmed that saturation was met [9]. GP characteristics were 5 females and 5 males, and with a mean age of 51 years ranging from 38 to 69 years. The GPs’ patient recruitment area was rural for 6 and urban for 4 of the GPs; see Multimedia Appendix 1.

### Interview Procedure

The semistructured interviews followed an interview guide, which allowed an iterative approach, where emerging themes and perspectives could be explored in the interviews with subsequent participants [10]; see Table 1 for an overview of the themes and probing questions in the interview guide. The interview guide was made with inspiration from a study on GPs attitude toward the treatment of cardiovascular disease [11] and a previous study exploring the patients’ perspective of using eHealth in changing lifestyle [8].

The interviews were held in Danish and carried out from March to May 2017 in the GPs’ offices and took 45-60 min each. All interviews were performed by CJB, who has worked as a GP for more than 10 years and with different eHealth solutions for more than 15 years. The GPs were asked to describe examples from their own experience and were encouraged to reflect upon them to explore the various aspects of the topics evolving.

### Analysis

The 10 interviews were digitally recorded and transcribed verbatim. The transcripts were analyzed by the researchers (CJB, GIS, JC, JBN, and JS) using thematic analysis. To systematically uncover important themes and to get a rich, straightforward description of the concepts and latent variables, the explorative approach of systematic text condensation was applied [12,13]. The transcripts were read thoroughly to get an overall impression of the material before the initial coding. A priori coding was done by using QSR NVivo 11 software [14] for each transcript. Themes were identified, and the data were coded, sorted, and categorized into themes and subthemes by identifying similar expressions, patterns, and sequences. Data from each theme were condensed and summarized into generalized descriptions and concepts concerning GPs’ perspectives on the use of eHealth in relation to improving lifestyle for their patients and themselves. During the analytical process, the extracted information was related to the full transcripts to preserve the original context. The identified themes were compared between the different researchers. Coinciding themes of importance was identified and consistency was reached.

**Table 1:** Interview guide. GP: general practitioner.

Themes	Probing questions
Experience with handling of patients with lifestyle challenges in their GP center	I will ask you to remember one consultation that went well. Please describe the consultation. What happened? What went well? What do you think a patient would choose given the choice between one pill or lifestyle change involving 30 min more daily exercise, healthier diet, and smoking cessation?
Their own lifestyle experiences	Have you ever taken the initiative to improve or change your lifestyle? Who has helped you with your health challenges?
Experience with eHealth in relation to their own and patients’ health challenges	Have you ever used apps or internet in relation to your own health or well-being? Have you communicated with your patients using digital tools? How? Have you ever received patient-registered objective measurements via digital tools? How do you use them?

Finally, quotes were selected to illustrate each theme and its related subthemes and translated from Danish to English. The two researchers, CJB and GIS, compared their individual translations, agreeing on wording and meaning. The remaining authors then reviewed the quotes in Danish and English, and changes were made if all authors agreed on it. In the text, interview quotes are followed by a unique participant identifier called GP1 to GP10 (Multimedia Appendix 1). The authors CJB and GIS were the only ones aware of the true identity of the GPs.

### Ethical Considerations

The study has been approved by the local Ethics Committee of Southern Denmark. Before initiating an interview with a GP, the nature of the research was briefly explained by CJB, any questions regarding the study were answered, and a description of the study in layman’s terms was provided. CJB explained that the interview data would be anonymized, the GPs were informed of their rights as participants, and informed consent documents were signed both by the GP and CJB.

## Results

### Themes Concerning Improving Lifestyle Using eHealth

We identified 3 themes with related subthemes concerning the use of eHealth in relation to improving lifestyle: (1) how eHealth is used for patients; (2) GPs own experience with improving lifestyle and eHealth support and (3) relevant coaching techniques for transformation into eHealth; see Table 2 for themes and related subthemes.

### How eHealth Is Used for Patients

All GPs used smartphones or tablets. All GPs used local electronic health record systems and asynchronous e-consultations daily related to exchange of laboratory results and simple health questions, which are embedded in the local health record system, but only 1 had experience with PROM delivered to the GP via eHealth solutions in the form of home-registered blood pressure. Most eHealth communication reported was one-way such as showing results on the GP’s computer screen, or the GP recommending websites and apps to the patient.

#### One-Way Information About Health and Lifestyle in the Consultation

A total of 9 out of the 10 GPs only used one-way information about health and lifestyle in the consultation. Typically the GPs used their computer screen when showing patients the development of objective risk factors/lifestyle measures such as HbA1c, cholesterol, or weight. One GP stated:

I use numbers and figures from my computer screen to explain to the patients how they are doing.

GP1

#### Recommendation of Websites That Patients Could Use on Their Own

Recommendation about eHealth for patients’ personal use most often consisted of websites with relevant information that they found matched the patient. One of the GPs said:

It could be concrete sleep hygiene advice and instructions. Either from me or if it is a younger person, from websites they can visit.

GP9

#### Recommendation of Apps That GPs Had Used Themselves or Learned About From Other Patients

In addition, 5 GPs recommended apps that they had used themselves or learned about from other patients:

Well, sometimes they bring something up. For example, the one called “7 Minute Workout”; I could recommend that one to some of my other patients, because the vast majority can do that…But, sometimes it is the patient, who tells me about something smart, which I also think is smart.

GP3

#### Attitude to Lifestyle Intervention, Use of eHealth, Workflow, and Data Security

All GPs underestimated their patients’ preference for lifestyle improvement over medicine. Most thought 50% would prefer medicine to lifestyle intervention. Most GPs were very positive when discussing how they could follow patients’ lifestyle through smartphones with step counts, etc:

If I had problems completing something that would improve my health and it could be supported broadly by electronics, apps or pulse clock or...then I think I would benefit from it.

GP6

**Table 2:** Themes and subthemes for general practitioners’ (GP) perception of electronic health (eHealth) in relation to lifestyle improvement.

Theme 1. How eHealth is used for patients	Theme 2. GPs own experience with improving lifestyle and eHealth support	Theme 3. Relevant coaching techniques for transformation into eHealth
One-way information about health and lifestyle in the consultation	Mirroring own personal health situation and procedural knowledge	Mutual understanding of patient challenges is key
Recommendation of websites that patients could use on their own	Realistic goal setting	Realistic goal setting
Recommendation of apps that GPs had used themselves or learned about from other patients	Measurable outcome and reinforcement	Measurable outcomes
Attitude to lifestyle intervention, use of eHealth, workflow, and data security	Support from family and peers	Social and structural barriers

Concerns were mainly aimed at how to integrate the eHealth data without disturbing other tasks that needed attention or exposing sensitive data:

I’m afraid it will take up too much time.

GP1

One GP expressed concerns related to security of data:

Is the data security good enough?

GP3

### General Practitioners’ Own Experience With Improving Lifestyle and eHealth Support

#### Mirroring Own Personal Health Situation

A total of 9 out of the 10 GPs said they wanted to live healthier than they did, and many explained how eHealth supported them in their daily healthy lifestyle choices:

Using a pedometer, while working in the clinic, we suddenly realized, that we actually walked less than we thought.

GP3

#### Realistic Goal Setting and Procedural Knowledge

One of the major barriers for the GPs was setting up realistic plans for themselves and following them. Some had used apps to support their health plan:

...fulfill the app´s needs in a way. In some way, it needs to know whether you have made your push-ups today, and then you get a need to say yes to it.

GP2

#### Measurable Outcome and Reinforcement

Most of the GPs explained how they valued being able to measure their progress and being recognized for their effort:

It is important to praise the patients...I need to be recognized in one way or another every time I exercise. If I forget my phone I don’t exercise.

GP4

#### Support From Family and Peers

Most GPs found themselves or their spouse to be the most important support for healthier living:

No, well the wife indirectly…And again, that is the competitive element that kicks in, you should not underestimate the value it has, if you are into that. I remember one day, when she worked late and we couldn’t walk the dog together as we usually do, then I was ahead of her stepwise (laughing). I enjoyed that because usually she lies ahead of me.

GP4

The majority of GPs would not share health improvement data on Facebook, but held positive viewpoints of sharing data online with other persons having the same health issues as themselves:

...a group, you just sign up for, it is about getting the support in order to live healthier, to be around someone, who has the same problem and then meet regularly.

GP2

**Relevant Coaching Techniques for Transformation** **Into** **eHealth**

All GPs used motivational interviewing in their communication with patients about lifestyle.

#### Mutual Understanding of Patient Challenges Is Key

All the GPs highly valued the relationship with the patient, and the majority found they needed to know the reasons behind lifestyle choices and that could only be learned from the patient:

...understanding, the patient’s starting point, getting to know a little more about their specific situation, and also getting to know their conceptual framework of different things.

GP6

Many of the GPs could explain how they had learned this from their own experience with changing lifestyle:

...but if you then hear someone, who talks about skinny-fat, eg, then you catch the message, right? Because then it becomes—what should I call it—something I can identify myself with…It becomes relevant to me.

GP4

...and we actually made motivational interviewing about exercise for each other, after which I also started cycling to work on a daily basis (laughing), and it also changed the way I looked at motivational interviewing, because I think it worked annoyingly good, also on me.

GP2

With regard to eHealth, for patients with chronic health issues who GPs knew were challenged with their health, eHealth was viewed as to not “loose them for follow-up”:

...we have, ie, chronic patients with hypertension, COPD, diabetes, and we need to have a waterproof system. A system to ensure that, when they are in the system, they don’t leave without a scheduled appointment, and if we catch them in an exacerbation (Editor’s note: if the patient’s condition worsens), then we get them back on the tracks again etc., but we don’t have a system, ie, this female patient, who cancelled an appointment and then she was lost again.

GP6

#### Realistic Goal Setting

The GPs found it important that it is the patients who set the goals:

It must be them, who are setting the goals. They have to have ownership, else it won’t work at all…what they are doing has to be of great importance for them, and it has to be what they find utmost important, and at the same time what they think they can complete and what they find realistic to integrate in their everyday life.

GP3

This was an experience they could relate to in different ways:

I have also tried to lose weight. It has helped a lot that I bought an electric bike, because then I get, then I am much more motivated to get out and go for a ride on the bike.

GP9

Furthermore, by using eHealth:

Well…You can see it on the watch, the way it looks, there is a number especially women, who wear accelerometers in watches and then I see, that it is an ongoing motivation, that they wear it, they walk more and go for extra walks consciously.

GP6

#### Measurable Outcomes

PROM data were used by all GPs in the form of paper notes brought to the GP by the patient to facilitate discussions in the consultation room:

Sometimes I think, and we have been doing that for many years, for example if it concerns such a thing as a weight loss, that I simply start out by giving them a paper, with a table drawn on it, covering all the week days, and then it says: breakfast, snack, lunch, snack, dinner. And then they simply have to fill in what they eat at all times, so that we can use it as a starting point.

GP5

And more specific with relevance for eHealth:

Yes, sure I do that (Editor’s note: use PROM) It is often blood glucose measurements (written on paper, red). Steps (information about steps. red.) could also be a possibility, but then it is more unspecific, there are not many who measure their function so specific, not among our patients anyway. Then it is more like: “I walk two kilometers so and so.” So it could very well be more specified, actually, I think. And then I would use it, then you actually could use it, if they could say: “Yesterday I walked exactly…” then you, as a GP, would use it if they came in with their measurements. Because often it gets very unspecific.

GP7

#### Social and Structural Barriers

It was important for all GPs to get to know their patients and learn what barriers they experienced in their lives that prevented them from making lifestyle choices that were most healthy for them. Many explained how they helped the patient to find possibilities in their daily life to fit in more exercise:

It is not something you have to decide in the evening, Monday evening, whether you want to go out for a spinning hour or not. It is more like, now I leave work to go home, I do not have a ticket for the bus, so now I walk home. Or, now I am going home from work, and my bike is outside, so now I am riding the bike home.

GP2

GPs, especially those with many years of experience, found that some life events were so important to patients that lifestyle change became inevitable:

Patients with myocardial infarction are easy to assist in smoking cessation.

GP8

Actually, sometimes I am surprised by how much people are capable of changing their lifestyle because they are getting diagnosed.

GP9

Some GPs pointed out that eHealth might be a way to support collaborations with lifestyle coaching specialists:

I think if we had some places we could refer them to, or someone who came to the clinic and offered exercise, diet counseling and such things, because in a regular consultation where we take care of the medicine and everything else about the disease and its consequences, eyes, legs, then there is not much time left for other things that really fill up.

GP10

## Discussion

### Principal Findings

eHealth solutions were generally not used when communicating with patients, and if used, they were used only as one-way recommendations from GP to patient; however, they play an important role for the majority of the GPs’ own lifestyle choices. Furthermore, our study showed that GPs used motivational interviewing, were positive to new technology, and gave many insights into how coaching techniques could be included in their patient communication for lifestyle improvement.

### Comparison With Prior Work

#### eHealth Use for Patient Communication

Our finding that GPs only used eHealth one way is in alignment with a recent study including interviews with 3 GPs and 9 other health care professionals, demonstrating that GPs and health care professionals most often used eHealth by recommending websites, even though they saw eHealth enabling patients to participate in balanced two-way conversations in face-to-face consultations [15].

The lack of more advanced eHealth use can both be due to patients’ preconception of what they expect from their GP as well as GPs’ reluctance in using new technology. Bowes et al [16] found that patients who had found information on the internet prioritized the opinion from their doctor higher than information found on the internet, except when the doctors were disinterested, dismissive, or patronizing. Then, the doctor-patient relationship was damaged, and patients would seek information from alternative routes. Creating space for eHealth in GPs’ patient communication is a two-way street. A Danish study suggests that GPs have a fundamental different perspective of what digital interaction can be used for compared with patients’ perspective. Patients often expected a dialogue with room for discussion, whereas GPs mostly saw e-consultation as a tool for short closed information, which might be due to a lower remuneration for e-consultations for the GPs compared with face-to-face consultations [17]. Furthermore, GPs often have a strong relationship with their patients, which we anticipate could be of importance for developing new collaborating eHealth solutions, where adherence to agreed treatment based on an existing relationship is often an issue [18]. Studies have shown that quality of both websites and apps vary [16,19], which might also play a role in the GPs lack of eHealth use in addition to the GPs explaining that it was difficult to know what to recommend. Generally, however, along with other studies, we also found that GPs as health professionals are positive to the use of eHealth solutions [15,20]. All GPs in our study underestimated the patients’ wish for lifestyle advice [1]. One of the main challenges seems to be to fit eHealth and lifestyle talks into the known workflow, highlighting that competing priorities might be one of the major obstacles [15,21]. Asynchronous e-consultations have been used for more than 10 years by GPs in Denmark, and concerns of security of data were only raised by one GP, a concern that seemed to be more prominent in other studies [22].

#### GPs’ Personal Experience Improving Lifestyle and eHealth

GPs recognized the positive effects of wearables, apps, and internet for their own personal health and how it animated them to live healthier in accordance with known behavioral change theories [23]. GPs found that eHealth monitoring through measurable outcomes helped them to set realistic goals and reminded them of how even small steps could help them live healthier in accordance with studies looking into a number of behavioral change techniques used in eHealth [24]. GPs also explained how patients gave them ideas to use different apps for benefiting their own health. A trend that was also noted in an English study on the role of GPs, finding that this could be understood in an Eliasian framework as the functional-democratization of patient-doctor relations via civilizing processes [25]. As a GP, you have to take up different roles. An Australian study from 2016 has described how GPs on help lines not being able to see patients face to face have to take a new role [26]. A total of 9 out of the 10 GPs wanted to live healthier, and eHealth solutions gave the GPs the opportunity as private persons to live healthier. GPs experienced that being supported both from family and peers mattered. Maybe GPs when discussing lifestyle with patients should take a new role and discuss GPs as human beings also have to make lifestyle choices on a daily basis to stay healthy, mainly because it opens for a respectful empathic dialogue, which is important for patients’ long-term successful lifestyle improvement [8].

#### Future Use of eHealth in Patient Lifestyle Coaching

Transforming knowledge into action is not trivial, and the GPs were positive in regard to empowering patients through increasing the patient’s capacity to think critically and make autonomous, informed decisions, which is in accordance with Anderson and Funnell [27], but contrary to another recent study where GPs expressed nervousness for patients performing self-care [28]. The GPs told how they used coaching techniques such as motivational interviewing to set realistic goals, focusing on measurable outcomes, overcoming structural barriers, and having patients commit to concrete realistic contracts using pen and paper [23]. GPs expressed the will to assist patients in healthy lifestyle choices and deep reflection monitored through PROM delivered via eHealth. Studies show that PROM data could be supported by algorithms based on eHealth [29] and through patient-centered care [30,31], giving space for the patient to act. Adding machine learning or artificial intelligence to PROM data could potentially strengthen the GPs’ ability to assist patients cost efficiently in conjunction with other health care professionals, which is being tested in specific patient groups [32], but will need substantially more research.

### Strengths and Limitations of This Study

This is the first qualitative research study to analyze how GPs using eHealth perceive eHealth in relation to successful lifestyle change among patients and themselves. However, even though the findings of this study are relevant and seem generalizable to future implementations of eHealth solutions involving GPs, it will not be applicable to all health care systems.

A limitation of this study is also the lack of methodological triangulation: why further studies using questionnaires and more quantitative outcomes are needed. Another limitation is that we only interviewed GPs. Data from patients could have revealed other aspects of the GPs’ role in assisting patients’ lifestyle change.

### Conclusions

GPs used eHealth for their own health but did not translate that into lifestyle change guidance for their patients, although they had been inspired themselves from discussions with patients. eHealth has the potential to become an important tool for the GPs in future work to improve the health of their patients. Education is needed, remuneration structures may need to be revisited, and more research is needed on how GPs can become active in developing behavioral change eHealth solutions that will create the future framework for collaboration among general practice, local authorities, and patients.

## Appendix

Multimedia Appendix 1 General practitioner characteristics.

## Abbreviations

eHealth: electronic health
GP: general practitioner
PROM: patient-reported outcome measures

## References

[1] Jarbøl DE, Larsen PV, Gyrd-Hansen D, Søndergaard J, Brandt C, Leppin A, Barfoed BL, Nielsen JB (2017). Determinants of preferences for lifestyle changes versus medication and beliefs in ability to maintain lifestyle changes. A population-based survey. Prev Med Rep.

[2] Hermans M, Van Gaal L, Rézette I, Daci E, MacDonald K, Denhaerynck K, Vancayzeele S, Dej Meester L, Clemens A, Yee B, Abraham I (2016). Patient engagement impacts glycemic management with vildagliptin and vildagliptin/metformin (single pill) regimens in type 2 diabetes mellitus (the GLORIOUS study). Prim Care Diabetes.

[3] Afshin A, Babalola D, Mclean M, Yu Z, Ma W, Chen CY, Arabi M, Mozaffarian D (2016). Information technology and lifestyle: a systematic evaluation of internet and mobile interventions for improving diet, physical activity, obesity, tobacco, and alcohol use. J Am Heart Assoc.

[4] Levine DM, Savarimuthu S, Squires A, Nicholson J, Jay M (2015). Technology-assisted weight loss interventions in primary care: a systematic review. J Gen Intern Med.

[5] The Ministry of Health (2017). Healthcare in Denmark - An overview.

[6] Brandt V, Brandt CJ, Glintborg D, Arendal C, Toubro S, Brandt K (2011). Sustained weight loss during 20 months using a personalized interactive internet based dietician advice program in a general practice setting. International Journal on Advances in Life Sciences Internet.

[7] Haste A, Adamson AJ, McColl E, Araujo-Soares V, Bell R (2017). Web-based weight loss intervention for men with type 2 diabetes: pilot randomized controlled trial. JMIR Diabetes.

[8] Brandt CJ, Clemensen J, Nielsen JB, Søndergaard J (2018). Drivers for successful long-term lifestyle change, the role of e-health: a qualitative interview study. BMJ Open.

[9] Mason M (2010). Sample Size and Saturation in PhD Studies Using Qualitative Interviews. FQS.

[10] Kvale S, Brinkmann S (2014). InterViews: Learning the Craft of Qualitative Research Interviewing.

[11] Barfoed BL, Jarbøl DE, Paulsen MS, Christensen PM, Halvorsen PA, Nielsen JB, Søndergaard J (2015). GPs' perceptions of cardiovascular risk and views on patient compliance: a qualitative interview study. Int J Family Med.

[12] Sandelowski M (2000). Whatever happened to qualitative description?. Res Nurs Health.

[13] Malterud K (2001). Qualitative research: standards, challenges, and guidelines. Lancet.

[14] (2017). QSR International.

[15] Macdonald GG, Townsend AF, Adam P, Li LC, Kerr S, McDonald M, Backman CL (2018). eHealth technologies, multimorbidity, and the office visit: qualitative interview study on the perspectives of physicians and nurses. J Med Internet Res.

[16] Bowes P, Stevenson F, Ahluwalia S, Murray E (2012). 'I need her to be a doctor': patients' experiences of presenting health information from the internet in GP consultations. Br J Gen Pract.

[17] Hansen CS, Christensen KL, Ertmann R (2014). Patients and general practitioners have different approaches to e-mail consultations. Dan Med J.

[18] Lie SS, Karlsen B, Oord ER, Graue M, Oftedal B (2017). Dropout from an eHealth intervention for adults with type 2 diabetes: a qualitative study. J Med Internet Res.

[19] Gibbs J, Gkatzidou V, Tickle L, Manning SR, Tilakkumar T, Hone K, Ashcroft RE, Sonnenberg P, Sadiq ST, Estcourt CS (2017). 'Can you recommend any good STI apps?' A review of content, accuracy and comprehensiveness of current mobile medical applications for STIs and related genital infections. Sex Transm Infect.

[20] Sprenger M, Mettler T, Osma J (2017). Health professionals' perspective on the promotion of e-mental health apps in the context of maternal depression. PLoS One.

[21] King G, O'Donnell C, Boddy D, Smith F, Heaney D, Mair FS (2012). Boundaries and e-health implementation in health and social care. BMC Med Inform Decis Mak.

[22] Bellicha A, Macé S, Oppert JM (2017). Prescribing of electronic activity monitors in cardiometabolic diseases: qualitative interview-based study. J Med Internet Res.

[23] Bandura A (2004). Health promotion by social cognitive means. Health Educ Behav.

[24] Lyons EJ, Lewis ZH, Mayrsohn BG, Rowland JL (2014). Behavior change techniques implemented in electronic lifestyle activity monitors: a systematic content analysis. J Med Internet Res.

[25] Brown P, Elston MA, Gabe J (2015). From patient deference towards negotiated and precarious informality: An Eliasian analysis of English general practitioners' understandings of changing patient relations. Soc Sci Med.

[26] McKenzie R, Williamson M (2016). The league of extraordinary generalists: a qualitative study of professional identity and perceptions of role of GPs working on a national after hours helpline in Australia. BMC Health Serv Res.

[27] Anderson RM, Funnell MM (2010). Patient empowerment: myths and misconceptions. Patient Educ Couns.

[28] Scambler S, Newton P, Asimakopoulou K (2014). The context of empowerment and self-care within the field of diabetes. Health (London).

[29] Wells S, Kerr A, Broadbent E, MacKenzie C, Cole K, McLachlan A (2011). Does your heart forecast help practitioner understanding and confidence with cardiovascular disease risk communication?. J Prim Health Care.

[30] Collins C, Rochfort A, Capelli O (2016). Promoting Self-Management and Patient Empowerment in Primary Care. Primary Care in Practice - Integration is Needed.

[31] Van der Heide I, Snoeijs SP, Boerma WGW, Schellevis FG, Rijken MP, Richardson E, Van Ginneken E (2016). How to strengthen patient-centredness in caring for people with multimorbidity in Europe?. Health Systems and Policy Analysis: Policy Brief 22.

[32] Piette JD, Krein SL, Striplin D, Marinec N, Kerns RD, Farris KB, Singh S, An L, Heapy AA (2016). Patient-Centered Pain Care Using Artificial Intelligence and Mobile Health Tools: Protocol for a Randomized Study Funded by the US Department of Veterans Affairs Health Services Research and Development Program. JMIR Res Protoc.

