# Supplementary material for: General Practitioners’ Perspective on eHealth and Lifestyle Change: Qualitative Interview Study
Source: JMIR Mhealth Uhealth. 2018 Apr 17;6(4):e88. doi: 10.2196/mhealth.8988 (PMC5930171; doi:10.2196/mhealth.8988)
Supplement: Multimedia Appendix 1 [file mhealth_v6i4e88_app1.pdf]

Appendix 1. GP characteristics

Participant ID	Gender	Age	Type of Practice	Patient recruitment area
GP1	Male	54	Solo practice	Urban
GP2	Female	46	Solo practice	Rural
GP3	Female	53	Shared practice	Rural
GP4	Male	45	Shared practice	Urban
GP5	Female	48	Shared Practice	Rural
GP6	Male	51	Shared practice	Rural
GP7	Female	38	Shared practice	Rural
GP8	Male	69	Solo practice	Rural
GP9	Male	65	Shared practice	Urban
GP10	Female	39	Shared practice	Urban
